# Pasture Access Affects Behavioral Indicators of Wellbeing in Dairy Cows

**DOI:** 10.3390/ani9110902

**Published:** 2019-11-01

**Authors:** Andrew Crump, Kirsty Jenkins, Emily J. Bethell, Conrad P. Ferris, Gareth Arnott

**Affiliations:** 1 Institute for Global Food Security, School of Biological Sciences, Queen’s University Belfast, 1-33 Chlorine Gardens, Belfast BT9 5AJ, UK; kjenkins04@qub.ac.uk (K.J.); g.arnott@qub.ac.uk (G.A.); 2 Research Centre in Brain and Behaviour, School of Natural Sciences and Psychology, Liverpool John Moores University, James Parsons Building, Byrom Street, Liverpool L3 3AF, UK; E.J.Bethell@ljmu.ac.uk; 3 Agri-Food and Biosciences Institute, Large Park, Hillsborough BT26 6DR, UK; Conrad.Ferris@afbini.gov.uk

**Keywords:** animal welfare, behavioral synchrony, confinement, continuous housing, lying, pasture, zero-grazing

## Abstract

**Simple Summary:**

Dairy cows in Europe and the United States are increasingly housed indoors year-round. Even cows with pasture access are usually kept inside during the winter and around calving. However, animal welfare scientists and dairy consumers are concerned that full-time housing impacts cattle welfare. We investigated how pasture influences behavioral indicators of wellbeing. Using cow pedometers, we recorded 29 animals’ lying and walking activity during 18 days of pasture access and 18 days of indoor housing. Cattle at pasture had fewer lying bouts but longer lying times, indicating they were more comfortable and less restless. Lying behavior was also more synchronous outdoors, with most of the herd lying at the same time. These results indicate pasture provides a comfortable surface and reduces competition for lying space. Furthermore, cows at pasture walked farther, with potential benefits for their physical health and psychological wellbeing. Our findings contribute to the growing body of evidence that pasture access improves dairy cow welfare. As a society, we must decide whether full-time housing is a price worth paying for dairy products.

**Abstract:**

Dairy cows are increasingly housed indoors, either year-round or for long stretches over the winter and around parturition. This may create health and welfare issues. In cattle, lying and walking are highly motivated, and herds synchronize lying behavior when they have comfortable surfaces and little competition for space. Lying and walking activity can, therefore, indicate good welfare. Using a repeated measures crossover design, we gave 29 Holstein–Friesian dairy cows 18 days of overnight pasture access (PAS treatment) and 18 days of indoor housing (PEN treatment). Accelerometers recorded their lying and locomotory behavior. We measured behavioral synchrony with Fleiss’ Kappa and analyzed the accelerometry data using linear mixed models. Compared to the PEN treatment, the PAS treatment had longer overnight lying durations (*χ*^2^_1_ = 27.51, *p* < 0.001), fewer lying bouts (*χ*^2^_1_ = 22.53, *p* < 0.001), longer lying bouts (*χ*^2^_1_ = 25.53, *p* < 0.001), and fewer transitions up or down (*χ*^2^_1_ = 16.83, *p* < 0.001). Herd lying behavior was also more synchronous at pasture (*χ*^2^_1_ = 230.25, *p* < 0.001). In addition, nightly step counts were higher in the PAS treatment than the PEN treatment (*χ*^2^_1_ = 2946.31, *p* < 0.001). These results suggest pasture access improves dairy cow welfare by increasing comfort, reducing competition and boredom, and facilitating motivated behavior.

## 1. Introduction

As global consumer demand grows, dairy farming will continue to intensify [1]. Housing cattle indoors year-round reduces labor inputs, facilitates the provision of high-energy diets, and increases milk yield without increasing farm size [2,3]. Cows in indoor housing are also better protected against endoparasites [4] and inclement weather [5]. As a result, the percentage of European and North American dairy cattle with pasture access is decreasing [6,7]. Across Europe, there is substantial variation in management. An estimated 98% of Irish and 92% of British dairy farms operate pasture-based systems, compared to only 20% in Czechia, less than 10% in Greece, and virtually none in Bulgaria [6]. In the United States, just 34% of dry cows and 20% of lactating cows are let out to pasture [7]. Even herds with pasture access are usually housed indoors over the winter and around calving.

However, full-time housing raises animal welfare concerns [8,9,10]. Compared to pasture, substrates tend to be more abrasive for lying and locomotion. Indoor housing is a risk factor for hock lesions [2], lameness [11,12,13], and mastitis [14,15], as well as injuries from slippage on slurry-covered concrete [16]. These health issues are painful for cattle [17,18] and may contribute to higher mortality in herds without pasture access [19,20,21,22]. In terms of behavior, indoor housing restricts movement and limits cows’ behavioral repertoire [23], potentially preventing the expression of motivated behaviors. Preference testing indicates that cattle given the choice spend longer at pasture, especially at night [24,25,26,27,28], although this effect may be reversed for animals reared indoors [29]. In motivation tests, cows are prepared to incur a cost for pasture access, such as walking long distances [30,31] or pushing weighted doors [32]. Consumers also value the perceived welfare benefits of pasture-based systems [33,34,35].

Lying behavior is a key indicator of cow welfare [36,37]. In dairy cattle, lying is highly motivated [38,39,40] and lying deprivation activates the hypothalamic–pituitary–adrenal axis [41,42,43]. Furthermore, rumination occurs whilst lying, so shorter lying durations jeopardize metabolic processes [44]. Disrupted lying behavior is also associated with lameness [45], mastitis [46], and enteritis [47]. Pasture is usually more comfortable than cubicles, with several studies finding longer lying durations at pasture than in indoor housing [12,48,49]. However, some researchers have reported longer lying times indoors [50]. This may reflect different activity budgets in indoor housing compared to pasture (e.g., reduced feeding durations), greater cow comfort in cubicles (e.g., by providing soft lying surfaces), or reduced cow comfort (e.g., due to difficulty standing) [9]. More consistently, dominant cows displace subordinates from cubicles [48,51]. Indoor housing, thus, reduces both total lying duration and mean lying bout duration, but increases the number of lying bouts [12]. This disrupted lying behavior indicates discomfort and competition for lying space.

As well as impacting lying activity, indoor housing desynchronizes herd behavior in dairy cows [51,52,53,54] and bulls [55]. Synchrony describes the proportion of individuals performing the same behavior at the same time. It occurs through two mechanisms: allelomimetic, when animals directly mimic conspecifics; and concurrent, when different individuals respond to the same cues in the same way [56]. As cows are herd animals, allelomimetic synchrony is internally motivated regardless of concurrent motivations, such as group milking and feeding [52]. Synchrony is, therefore, a characteristic of cows in semi-natural environments, including pasture [52,57]. Desynchronization is linked to reduced lying time, more cubicle displacements, and more daytime lying in subordinate individuals [58,59,60]. Consequently, many authors have suggested synchrony signals good welfare [10,51,61,62,63,64]. As with less disrupted lying behavior, synchrony reflects reduced competition and natural behavior patterns.

Locomotion also increases at pasture [24,29,50,53,65]. During grazing, cows spend more time walking than when they are feeding indoors, and grazing areas are normally farther from the milking parlor than the feeding area of indoor housing. Walking is a “behavioral need”; cows are motivated to walk even without external motivations. Krohn et al. [53] gave dairy cattle free access to indoor and outdoor areas. Despite having food, water, and shelter inside, subjects walked outside for 2.5 km per day in summer and 0.8 km per day in winter. Moreover, cows, calves, and heifers that spend longer indoors are more active after being let out [66,67,68]. These findings suggest exercise is a positive welfare outcome in itself. Walking also has physical benefits, especially for cows’ legs, feet, and hooves [50,68,69,70]. Gustafson and Lund-Magnussen [71] suggested exercise improves the condition of joints, tendons, and ligaments in dairy cows, easing transitions up and down. Regular walking on a treadmill reduced gestating cows’ working heartrate and plasma lactate concentrations, indicating reduced metabolic stress [72]. Therefore, higher step counts improve health, as well as demonstrating increased grazing if cows have access to pasture.

To date, limited research has investigated the welfare impacts of restricted pasture access, where cows are fed indoors for part of the day [26,73]. Moreover, few experimental studies have compared behavioral welfare indicators at pasture and in indoor housing [8]. Using a repeated measures crossover experiment, we addressed these gaps by recording dairy cows’ lying and walking activity during 18 days of overnight pasture access and 18 days of full-time housing. We predicted that cows at pasture would have longer total lying durations, fewer and longer lying bouts, more synchronous lying behavior, and higher step counts. These results would suggest pasture access improves dairy cattle welfare.

## 2. Materials and Methods 

### 2.1. Ethics

This study was approved by Queen’s University Belfast’s School of Biological Sciences Animal Research Ethics Committee (approval number: QUB-BS-AREC-18-005). In accordance with the Animals (Scientific Procedures) Act 1986, experimental procedures were described to a Home Office inspector beforehand and deemed not to require a license. Animal welfare was prioritized throughout.

### 2.2. Subjects and Housing

This study was carried out during summer 2018 at the Agri-Food and Biosciences Institute (AFBI), Hillsborough, County Down, Northern Ireland (54°5’ N; 6°1’ W). The experiment involved 29 autumn-calving, lactating, Holstein–Friesian dairy cows (mean of 4.34 years, range 2.69–8.72 years; mean of 241 days calved, range 209–273 days). We performed a power analysis to determine sample size; an effect size of 0.5, significance level of 0.05, and power of 0.8 required 27 individuals. All subjects were kept at pasture prior to the study, but they were housed inside for eight weeks pre-testing to standardize conditions (see below). The indoor housing consisted of two adjoining pens (each 13.3 × 8.5 m). Both pens had 16 cubicles, fitted with rubber mats, and concrete standing and walking areas, cleaned by an automatic scraper system six times per day. The building was naturally ventilated, with no additional ventilators servicing the pens. Cows had ad libitum access to grass silage offered daily at approximately 09:00 via an open feed barrier along the front of each pen, and ad libitum access to fresh water. They were milked in a rotary parlor twice daily (06:30 and 15:00).

Before the experiment, all 29 subjects were fitted with an IceTag (IceRobotics Ltd., Edinburgh, UK). IceTags are commercially available hind-leg activity monitor sensors that distinguish lying from standing and record step counts using a tri-axial accelerometer (sampling rate: 16 Hz; time resolution: 1 s; dimensions: 95.0 × 82.3 × 31.5 mm; weight: 130 g; validated by [74,75,76,77]). As well as the study animals, the herd included three cows whose behavior was not recorded, due to lack of IceTag availability (total herd size: 32). These three additional animals allowed us to maintain a consistent 1:1 cow/cubicle ratio. Four days before testing, a veterinary graduate scored each subject’s mobility, following a standard four-point system developed by the Agriculture and Horticulture Development Board [78] (Table 1). Cattle were individually observed from the front and side, whilst walking and standing on a flat surface. Scores of 0 or 1 were classified as non-lame; scores of 3 or 4 were classified as lame (for results, see Table 1).

### 2.3. Procedure and Treatments

Before the study, all 32 cows were housed in the indoor pens without pasture access for eight weeks. The pens were connected and the animals were managed as one group. When the experiment began, cows were pseudorandomly divided into two groups of 16 (balanced for lameness) and the pens were visually isolated from each other using plywood sheeting. The experiment used a two-period crossover design with two concurrent treatments: 18 days of overnight pasture access (PAS) and 18 days of full-time housing (PEN) (first period: June 25, 2018–July 13, 2018; second period: July 16, 2018–August 03, 2018). Throughout the study, both groups were kept in the indoor pens with the same silage type from 10:00 to 16:00. Cows in the PEN treatment were also housed overnight, with ad libitum access to silage. Cows in the PAS treatment had 18 h of daily pasture access, from approximately 16:00 (post-afternoon milking) until 10:00 the next morning. This covered the main grazing times (dawn and dusk [26,73,79,80]) and is when cattle choose to access pasture [24,25,26,27,30,31]. PAS cows were managed in a rotational grazing system, so the treatment groups were kept on different pastures. Area grazed ranged 1370–3950 m^2^ and distance to parlor ranged 190–295 m. We analyzed grass samples three times during each period (six times in total). The herbage was generally high quality, although lower quality in the second period. Across the study, mean oven dry matter (DM) content was 226.8 (SD 27.8) g/kg, mean crude protein content was 216.5 (SD 24.2) g/kg DM, and mean metabolizable energy content was 11.4 MJ/kg DM (first period: 238.5, SD 8.6, g/kg; 226.0, SD 11.5, g/kg DM; and 12.0 MJ/kg DM, respectively; second period: 215.0, SD 8.6, g/kg; 207.0, SD 11.5, g/kg DM, 10.9 MJ/kg DM, respectively). When the first period was complete, the cows swapped treatments and the procedure was repeated. The group at pasture first (PAS-first) had 14 study animals (mean of 4.47 years, range 2.69–8.72 years; mean of 240 days calved, range 219–260 days) and the group at pasture second (PAS-second) had 15 study animals (mean of 4.22 years, range 2.74–7.76 years; mean of 242 days calved, range 209–273 days). Including the three individuals without IceTags, both groups contained 16 cows in total. As part of a larger project, other welfare measures were collected during daytime housing, including performance in cognitive bias tasks, eye temperature, and health scores [81].

### 2.4. Data Preparation

Using the IceTags, we measured seven variables: overnight lying duration (h/night), daytime lying duration (h/daytime), number of lying bouts (bouts/24 h), lying bout duration (total duration/bouts), overnight transitions up or down (transitions/night), daytime transitions (transitions/daytime), and overnight step count (steps/night). Overnight data were analyzed from 16:30 to 09:30 and daytime data from 10:00 to 15:00, so effects of walking to and from pasture were eliminated. Lying duration was the total time the IceTag was horizontal, lying bouts were the duration from vertical to horizontal and back again, and steps were counted whenever cows lifted their tagged leg. Lying duration, transitions, and step counts were recorded in 15-min intervals; bout length data were only available per day. To measure synchrony, we classified cows as lying if they spent over half the 15-min interval lying (>449 s). We compared the binary lying data (either lying or not) between herd members within each interval. To our knowledge, this automated method is a novel way to assess behavioral synchrony (further detailed below).

### 2.5. Statistical Analyses

We analyzed the data in R (R Core Team, Cran-r-project, Vienna, Austria, version 3.4.4). Data were checked for normality by plotting histograms; transformations were applied where these improved the distribution. We fitted general linear mixed effects models (GLMMs) using maximum likelihood (ML), including all interactions. To improve the models’ fit to the data, we removed interactions in a stepwise fashion and selected models with the lowest Akaike information criterion (AIC) values. We re-ran these models using the restricted maximum likelihood (REML) approach. *P*-values were extracted using a Wald’s test, with *p* < 0.05 considered statistically significant. Data are presented as means ± standard error.

We fitted separate models for the following response variables: overnight and daytime lying duration, number of lying bouts, lying bout duration, overnight and daytime transitions, and overnight step count. The fixed effects were treatment, treatment order (either PAS first or second), and cow age (column scaled and centered in R), whilst cow ID and day number were random effects. Based on AIC values, we removed interactions from the models for overnight lying duration (treatment × treatment order × age, treatment × age, treatment order × age), number of lying bouts (treatment × treatment order × age, treatment × age, treatment order × age), overnight transitions (treatment × treatment order × age, treatment × age), daytime lying duration (treatment × treatment order × age, treatment × age, treatment order × age), and overnight step count (treatment × treatment order × age, treatment order × age). Lying bout data included substantial outliers: the longest was 14.25 h, but the second longest was 7.77 h. As both values were from the same individual on consecutive days, we ran the bout models on both the original dataset and data within two SD of the mean. This did not change the significance level of any results, so only the original dataset model is reported. Because overnight step counts were positively skewed, we applied a square-root transformation to these data. Step counts are provided alongside walking distance, based on a stride length of 1.5 m [82].

We measured lying synchrony using Fleiss’ Kappa coefficient of agreement (K_F_), a test of inter-observer reliability for >2 raters [83]. Treating each cow as a rater, we measured synchrony as intra-herd “agreement” in lying behavior during each 15-min interval [61]. K_F_ > 0 indicates agreement greater than chance, K_F_ = 0 indicates chance levels, and K_F_ < 0 indicates disagreement greater than chance. Fleiss’ Kappa assumes independent data [84], which we determined with the IceTags’ recordings of maximum bout lengths. However, given the outliers in the lying bout data, we defined maximum bout length as two SD above the mean (3.75 h). This provided five intervals per night (17:00–17:15, 21:00–21:15, 01:00–01:15, 05:00–05:15, 09:00–09:15). Using the “IRR” package in R (Various Coefficients of Interrater Reliability and Agreement), we calculated daily K_F_ values for each treatment group and analyzed them as the response variable in a GLMM (fixed effects: treatment and treatment order; random effect: day number).

## 3. Results

We collected data from 29 cows across 36 days. However, the IceTags did not record every study day or 15-min interval for every subject, reducing the number of measurement days (number of cows × number of study days) and measurement intervals (number of cows × number of study intervals) available for analysis. For both overnight and daytime lying duration, we collected data from all individuals for every day (1044 measurement days). Overnight lying durations were compiled from 70,429 measurement intervals (563 measurement intervals unrecorded) and daytime lying durations were compiled from 20,759 measurement intervals (121 measurement intervals unrecorded). We gathered data on lying bout frequency and duration from 1034 measurement days (106 measurement days unrecorded). To measure transitions, we collected data for all subjects from every study day (1044 measurement days). Overnight transition data came from 70,429 measurement intervals (563 measurement intervals unrecorded) and daytime transition data came from 20,759 measurement intervals (121 measurement intervals unrecorded). For lying synchrony, we calculated 36 herd K_F_ values for both groups, with 18 per herd per treatment. These scores were based on 5140 measurement intervals from individual cows (80 measurement intervals unrecorded). Finally, we extracted step counts from 70,429 measurement intervals (563 measurement intervals unrecorded).

**Figure 1:**
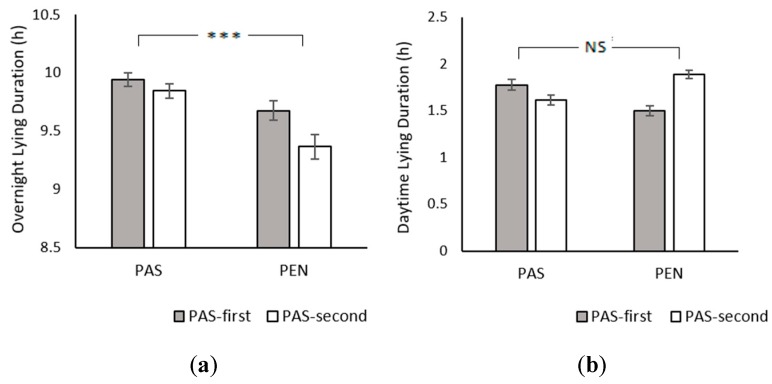
Effect of treatment and treatment order on (**a**) overnight lying duration and (**b**) daytime lying duration. Between-treatment significance levels: NS = non-significant; * = *p* < 0.05; ** = *p* < 0.01; *** = *p* < 0.001. Error bars represent the standard error of the mean. PAS = overnight pasture access; PEN = indoor housing.

Cows with pasture access had significantly longer overnight lying durations than cows indoors (PAS: 9.89 ± 0.04 h; PEN: 9.52 ± 0.07 h; *χ*^2^_1_ = 27.51, *p* < 0.001; Figure 1a). Neither treatment order (*χ*^2^_1_ = 0.94, *p* = 0.333), nor age (*χ*^2^_1_ = 0.24, *p* = 0.622), nor the treatment × treatment order interaction were significant (*χ*^2^_1_ = 2.21, *p* = 0.137). For daytime lying durations, there was no significant effect of treatment (PAS: 1.70 ± 0.04 h; PEN: 1.71 ± 0.04 h; *χ*^2^_1_ = 0.06, *p* = 0.814), treatment order (*χ*^2^_1_ = 0.41, *p* = 0.520), or age (*χ*^2^_1_ = 0.05, *p* = 0.824). There was a treatment × treatment order interaction (*χ*^2^_1_ = 41.88, *p* < 0.001; Figure 1b). The PAS-first group had longer daytime lying durations in the PAS treatment than the PEN treatment, but the PAS-second group had shorter daytime lying durations in the PAS treatment. In addition, the treatment × age interaction was marginally significant (*χ*^2^_1_ = 4.26, *p* = 0.039). Daytime lying times increased with age at pasture, but decreased with age in indoor housing. Neither the group × age interaction (*χ*^2^_1_ = 2.09, *p* = 0.148) nor the three-way interaction reached significance (*χ*^2^_1_ = 0.22, *p* = 0.641).

**Figure 2:**
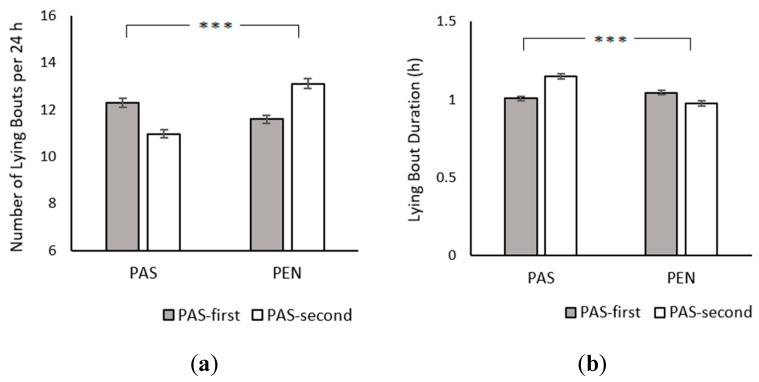
Effect of treatment and treatment order on (**a**) number of lying bouts per 24 h and (**b**) lying bout duration. Between-treatment significance levels: NS = non-significant; * = *p* < 0.05; ** = *p* < 0.01; *** = *p* < 0.001. Error bars represent the standard error of the mean.

PAS cows had fewer lying bouts than PEN cows (PAS: 11.65 ± 0.13; PEN: 12.31 ± 0.13; *χ*^2^_1_ = 22.53, *p* < 0.001; Figure 2a) and their lying bouts were significantly longer (PAS: 1.08 ± 0.01 h; PEN: 1.01 ± 0.01 h; *χ*^2^_1_ = 22.53, *p* < 0.001; Figure 2b). Treatment order did not influence either the number (*χ*^2^_1_ = 0.03, *p* = 0.871) or duration of lying bouts (*χ*^2^_1_ = 0.03, *p* = 0.871). Age also had no effect on bout frequency (*χ*^2^_1_ = 0.07, *p* = 0.788) or length (*χ*^2^_1_ = 0.07, *p* = 0.788). However, there were significant treatment × treatment order interactions for number (*χ*^2^_1_ = 97.02, *p* < 0.001) and duration of lying bouts (*χ*^2^_1_ = 97.02, *p* < 0.001). Both groups had more and shorter lying bouts in their first treatment.

**Figure 3:**
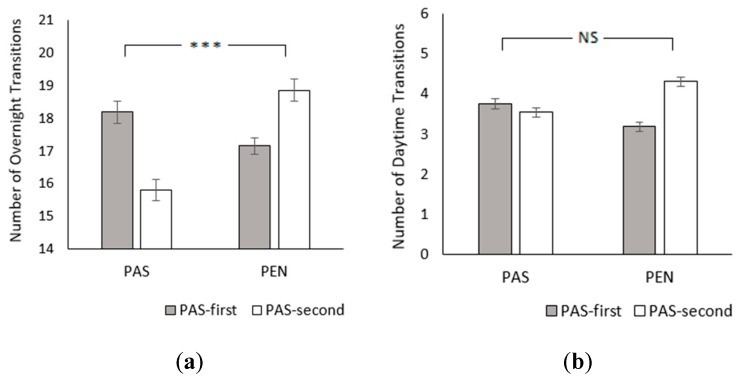
Effect of treatment and treatment order on (**a**) number of overnight transitions and (**b**) number of daytime transitions. Between-treatment significance levels: NS = non-significant; * = *p* < 0.05; ** = *p* < 0.01; *** = *p* < 0.001. Error bars represent the standard error of the mean.

There were significantly fewer overnight transitions in the PAS treatment than the PEN treatment (PAS: 16.96 ± 0.23; PEN: 18.04 ± 0.22; *χ*^2^_1_ = 16.83, *p* < 0.001; Figure 3a). Neither treatment order (*χ*^2^_1_ = 0.08, *p* = 0.775) nor age affected transition frequency (*χ*^2^_1_ = 0.32, *p* = 0.571). Nonetheless, there was a treatment × treatment order interaction (*χ*^2^_1_ = 55.71, *p* < 0.001). In the PAS-first group, subjects transitioned more at pasture than inside, whereas PAS-second cows had fewer transitions at pasture. There was also a significant treatment × age interactions (*χ*^2^_1_ = 12.65, *p* < 0.001); transition frequency increased with age in the PAS treatment and decreased with age in the PEN treatment. For daytime transitions, treatment (PAS: 3.65 ± 0.08; PEN: 3.76 ± 0.09; *χ*^2^_1_ = 1.37, *p* = 0.242), treatment order (*χ*^2^_1_ = 1.24, *p* = 0.265), and age were not significant (*χ*^2^_1_ = 0.01, *p* = 0.926), but the treatment × treatment order interaction persisted (*χ*^2^_1_ = 47.15, *p* < 0.001; Figure 3b).

**Figure 4:**
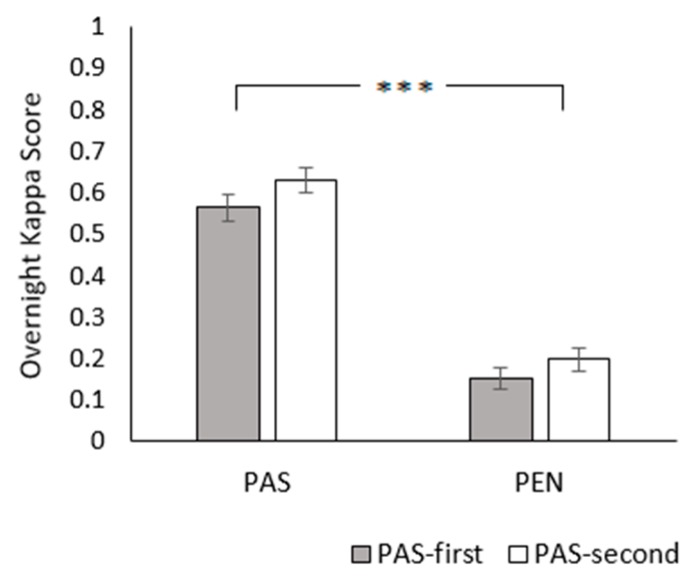
Effect of treatment and treatment order on overnight K_F_ (a measure of group synchrony). Between-treatment significance levels: NS = non-significant; * = *p* < 0.05; ** = *p* < 0.01; *** = *p* < 0.001. Error bars represent the standard error of the mean.

In terms of lying synchrony, K_F_ values were significantly greater in the PAS treatment than the PEN treatment (PAS: 0.60 ± 0.02; PEN: 0.18 ± 0.02; *χ*^2^_1_ = 230.254, *p* < 0.001; Figure 4). Treatment order also had a marginally significant effect, with lower K_F_ values in the PAS-first group (PAS-first: 0.36 ± 0.04; PAS-second: 0.41 ± 0.04; *χ*^2^_1_ = 4.007, *p* = 0.045). There was no treatment × treatment order interaction (*χ*^2^_1_ = 0.1628, *p* = 0.687).

**Figure 5:**
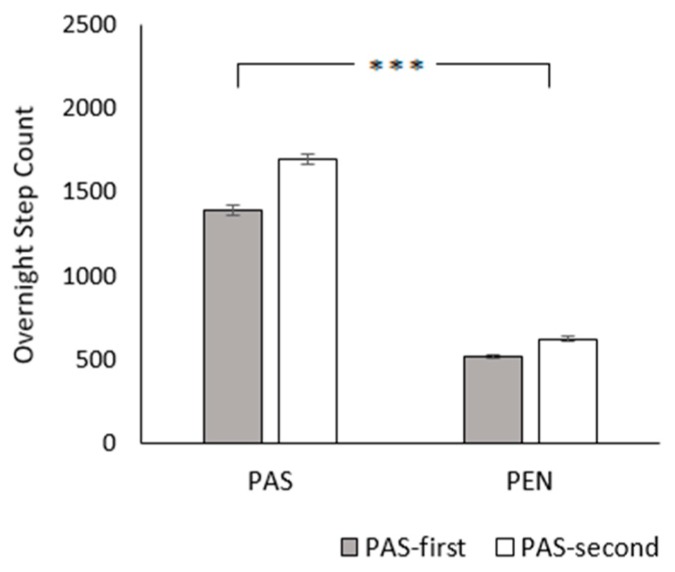
Effect of treatment and treatment order on overnight step count. Between-treatment significance levels: NS = non-significant; * = *p* < 0.05; ** = *p* < 0.01; *** = *p* < 0.001. Error bars represent the standard error of the mean.

Compared to the PEN treatment, overnight step counts were higher in the PAS treatment (PAS: 1548.45 ± 22.22 steps, 2.32 ± 0.03 km; PEN: 571.43 ± 9.76 steps, 0.86 ± 0.01 km; *χ*^2^_1_ = 2946.31, *p* < 0.001; Figure 5). There was a significant effect of treatment order, with lower step counts in the PAS-first group (PAS-first: 955.30 ± 24.65 steps, 1.43 ± 0.04 km; PAS-second: 1159.42 ± 29.01 steps, 1.74 ± 0.04 km; *χ*^2^_1_ = 9.68, *p* < 0.005). Step counts also decreased with age (*χ*^2^_1_ = 4.68, *p* = 0.031). Furthermore, the treatment × treatment order interaction was highly significant (*χ*^2^_1_ = 12.57, *p* < 0.001). PAS-first cows had a smaller increase in step count at pasture than PAS-second cows. The treatment × age interaction was also significant (*χ*^2^_1_ = 51.00, *p* < 0.001). At pasture, step counts decreased with age, whereas they increased with age indoors.

## 4. Discussion

We investigated how pasture access and indoor housing affect dairy cows’ lying and walking behavior as indicators of their welfare. In a two-period repeated measures crossover experiment, we compared activity between these two management systems using accelerometers. Pasture access increased behaviors associated with wellbeing in cattle and reduced signs of discomfort, displacements, and poor health. Overnight lying durations were longer at pasture, whilst there was no difference in daytime lying durations when both groups were in indoor housing. Lying is a highly motivated behavior which is important for cow welfare, so our results support previous work that cattle are more comfortable at pasture [38,39,40,41,43]. At pasture, cows also rested in fewer and longer lying bouts with fewer transitions and greater herd synchrony. This suggests pasture access reduces restlessness and competition for lying space [48,51]. Finally, cows had higher overnight step counts at pasture, likely because they spent more time grazing.

The lying data indicate that pasture provided a more comfortable surface than cubicles, and more lying space than fully-stocked indoor housing. Cows in the PAS treatment were less restless, with fewer but longer lying bouts, and fewer overnight transitions. Longer lying bouts reflect increased cow comfort [85]. Moreover, low-ranking individuals often cannot access cubicles at preferred times [12,48,49,58]. The treatment difference in overnight lying duration suggests additional lying bouts did not compensate for this disruption. In addition, we found no difference in lying duration or transitions during the daytime, when both treatments were housed indoors, implying pasture access was responsible. Our study also supports previous findings that pasture access increases herd synchrony in lying behavior [53,54,55]. Cattle synchronize under semi-natural conditions, indicating this is their preferred behavior pattern [52,57]. Whether animals have what they want is integral to welfare [86,87,88]. Our results suggest low-ranking cows in the PEN treatment could not lie when they wanted. Although cubicles were available for every animal, cattle exhibit longer lying durations, less daytime lying, fewer displacements, and greater lying synchrony when cubicle housing is understocked than fully-stocked [60]. This could be because limited cubicles prevent subordinates from lying where they want. Pasture, by contrast, provides ample lying space. As a result, we suggest pasture access promotes the animals’ agency, an important aspect of welfare [89].

Our findings also suggest boredom can be a welfare issue for cattle housed indoors. In animals, boredom is an aversive state that arises from general under-stimulation, rather than the frustration of any specific need or motivation [90]. Subjects in the PAS treatment spent a greater proportion of the night lying and walking, and cattle in the PEN treatment were standing inactive for longer. “Idle standing” may indicate poor welfare in cattle and is associated with hard lying surfaces [36,91,92]. From a health perspective, standing inactive can cause lameness, especially when the animal is partially in a cubicle [93,94,95], as well as being a symptom of disease (e.g., mastitis [96] or metritis [97]). Cows at pasture also spend a greater proportion of the day feeding [98], although the IceTags did not record these data. As such, cattle housed indoors have little to do for long timespans. Burn [99] linked under-stimulation with restlessness and disrupted sleep patterns in mammals (e.g., humans [100] and rats [101]). Boredom could, therefore, explain the PEN treatment’s disrupted lying behavior, compounded by abrasive surfaces and competition for cubicles. However, standing inactive has been attributed to depression-like states, as well as under-stimulation [102,103]. Isolating boredom requires specific behavioral indicators we did not record, such as measures of time perception and responses to novel stimuli [99,104].

Contrary to the overall lying results, both groups displayed signs of discomfort during the first testing period. The PAS-first group had longer daytime lying durations, more and shorter lying bouts, and more overnight transitions at pasture compared to indoor housing—results that were opposite to the PAS-second group. We attribute this to heat stress [17,105]. Despite similar mean daily temperatures in both periods, the maximum temperature was substantially higher in the first period (Table 2). Thermal stress reduces walking [17], which may be why the PAS-first group exhibited a smaller increase in step count at pasture than the PAS-second group. Moreover, daily sunlight hours were longer in the first period. Shade was not provided in the PAS treatment, further explaining the cows’ discomfort [5,106,107,108]. On the other hand, the first period had fewer hours per day with relative humidity ≥ 90%. Increasing relative humidity worsens heat stress [105]. During hot weather, some preference studies have recorded cattle spending more time in their indoor housing [25,27]. However, Charlton et al. [24] observed high temperatures increasing durations at pasture, possibly reflecting their setup’s temperate climate. This demonstrates the importance of context in dairy cow management. During extreme weather, pasture access may be detrimental to welfare if animals are forced to remain outside with no shelter.

The PAS treatment’s higher overnight step counts indicate that cows at pasture were healthier and satisfying a behavioral need, which indoor housing constrained. Previous studies have also found pasture access increases walking, because gait improves [50], feeding durations are longer [54,109], and cattle must continually walk whilst grazing [18]. Furthermore, treatment order had an effect, with less walking in the group that went out to pasture first. The increase in step counts in the PAS treatment was also smaller for the PAS-first group than the PAS-second group. This may reflect the higher quality herbage in the first period, which potentially reduced walking distances whilst grazing. Alternatively, PAS-first cows were indoors for 18 fewer days than PAS-second cows before going out to pasture. Longer indoor housing could have increased the PAS-second group’s motivation to move [66,67,68]. In addition to improving physical health, motor activity may enhance cows’ psychological wellbeing, as exercise can have antidepressant effects in humans [110,111,112,113]. To our knowledge, animal welfare scientists have not directly tested whether physical activity influences psychological indicators of wellbeing.

As lying and walking behavior indicated welfare was better when cows had access to pasture, we hope future research identifies the factors responsible [114]. This could lead to design and management practices that replicate the benefits of pasture in indoor housing [9]. For example, we linked restlessness to uncomfortable substrates and competition for cubicles. These issues can be partially addressed without pasture access. Tucker et al. [115] offered dairy cows three cubicle lying surfaces: deep-bedded sand, deep-bedded sawdust, and a rubber-filled mattress. Given the choice, subjects spent longer lying on sand and sawdust, and lying durations were shorter when only the mattress was available. Furthermore, Fregonesi et al. [58] reported that increasing stocking densities reduced lying durations and increased cubicle displacements. Fully or understocking cubicle housing can ameliorate this [60,116]. Additionally, enrichment could compensate for under-stimulating living conditions [117]. Brushes, for instance, increase total scratching time by over 500% in cubicle-housed cows, which may reduce boredom [118]. These findings indicate that cow welfare can be improved in indoor housing.

Nevertheless, going outdoors has health and welfare benefits, such as exposure to natural light [8]. Exercise yards have been proposed as an intensive alternative to pasture, because they require less space but allow cattle outside. However, cows with pasture access spend around twice as long outdoors compared to cows with exercise yards [26,119]. This indicates that pasture is preferred and implies that not all of pasture’s welfare benefits are transferable to more intensive production systems. Restricted pasture access, as in our study, may be a practical alternative [26,73]. Using the Welfare Quality ^®^ assessment protocol for dairy cattle [120], Wagner et al. [13] identified many of the same advantages for cows with 6–12 h of pasture per day as for cows with >12 h per day. Some features of indoor housing could also alleviate welfare issues at pasture, such as providing shade structures [5].

Finally, this study had several limitations. We did not control for grass intake in the PAS treatment, but diet influences cow behavior [121,122]. More generally, preference for pasture is modulated by factors both extrinsic (e.g., weather conditions [25,30]) and intrinsic to the cow (e.g., experience of pasture access [24,29]). Experiments such as ours are too small-scale to address whether the results apply to different herds on different farms at different times. Comparative observational studies are also necessary to understand how pasture and indoor housing influence welfare [13,123,124,125].

## 5. Conclusions

We performed a repeated measures crossover experiment to determine how overnight pasture access affects behavioral indicators of dairy cow welfare. As predicted, lying durations were longer at pasture than in indoor housing. Herd lying behavior was also more synchronous outside, and partitioned into fewer but longer lying bouts, with fewer transitions up or down. This suggests pasture was a more comfortable lying surface, reduced competition for lying space, and allowed cows to lie when and where they wanted. However, we found several unexpected treatment × treatment order interactions. Cows that went outside first were more restless at pasture than in indoor housing. We attribute this to heat stress and recommend providing shelter at pasture, depending on local climatic conditions. Additionally, overnight step counts were higher in the pasture treatment, which may benefit cattle physically and psychologically. Reduced lying and walking durations also suggest boredom is an issue in indoor housing, as cows have nothing to do for much of the day. Collectively, our results indicate overnight pasture access improves dairy cattle welfare.

## Figures and Tables

**Table 1:** Description of Mobility Scoring System, with baseline results for the present study (adapted from Reference [78]).

Score	Description of Cow Behavior	Classification	No. Subjects
0	Walks with even weight bearing and rhythm on all four feet, with a flat back; long, fluid strides possible	Non-lame	4
1	Steps uneven or strides shortened; affected limb or limbs not immediately identifiable	Non-lame	15
2	Uneven weight bearing on an immediately identifiable limb or obviously shortened strides (usually with an arched back)	Lame	8
3	Unable to walk as fast as a brisk human pace; lame leg easy to identify—limping; may barely stand on lame leg/s; back arched when standing and walking	Lame	2

**Table 2:** Meteorological data for both periods of the experiment (recorded 24 km from study site). Crown copyright (2018). Information provided by the National Meteorological Library and Archive–Met Office, United Kingdom.

Testing Period	Mean Temperature (°C)	Maximum Temperature (°C)	Sunshine Suration (h/d)	Relative Humidity ≥ 90% (h/d)	Rainfall (mm/d)
1	15.7	30.0	8.8	4.9	0.0
2	15.8	25.8	2.9	8.9	5.5
